# Phylogenetic analysis and a review of the history of the accidental phytoplankter, *Phaeodactylum tricornutum* Bohlin (Bacillariophyta)

**DOI:** 10.1371/journal.pone.0196744

**Published:** 2018-06-08

**Authors:** Jamal S. M. Sabir, Edward C. Theriot, Schonna R. Manning, Abdulrahman L. Al-Malki, Mohammad A. Khiyami, Areej K. Al-Ghamdi, Mumdooh J. Sabir, Dwight K. Romanovicz, Nahid H. Hajrah, Abdelfatteh El Omri, Robert K. Jansen, Matt P. Ashworth

**Affiliations:** 1 Genomic and Biotechnology Research Group, Department of Biological Sciences, Faculty of Science, King Abdulaziz University (KAU), Jeddah, Saudi Arabia; 2 Department of Integrative Biology, University of Texas at Austin, Austin, Texas, United States of America; 3 Department of Molecular Biosciences, University of Texas at Austin, Austin, Texas, United States of America; 4 Department of Biochemistry, Faculty of Science, King Abdulaziz University (KAU), Jeddah, Saudi Arabia; 5 King Abdulaziz City for Science and Technology, Riyadh, Saudi Arabia; 6 Department of Information Technology, Faculty of Computing and Information Technology, King Abdulaziz University (KAU), Jeddah, Saudi Arabia; 7 Center for Biomedical Research Support, University of Texas at Austin, Austin, Texas, United States of America; National Cheng Kung University, TAIWAN

## Abstract

The diatom *Phaeodactylum tricornutum* has been used as a model for cell biologists and ecologists for over a century. We have incorporated several new raphid pennates into a three gene phylogenetic dataset (SSU, *rbcL*, *psbC*), and recover *Gomphonemopsis* sp. as sister to *P*. *tricornutum* with 100% BS support. This is the first time a close relative has been identified for *P*. *tricornutum* with robust statistical support. We test and reject a succession of hypotheses for other relatives. Our molecular data are statistically significantly incongruent with placement of either or both species among the Cymbellales, an order of diatoms with which both have been associated. We believe that further resolution of the phylogenetic position of *P*. *tricornutum* will rely more on increased taxon sampling than increased genetic sampling. *Gomphonemopsis* is a benthic diatom, and its phylogenetic relationship with *P*. *tricornutum* is congruent with the hypothesis that *P*. *tricornutum* is a benthic diatom with specific adaptations that lead to active recruitment into the plankton. We hypothesize that other benthic diatoms are likely to have similar adaptations and are not merely passively recruited into the plankton.

## Introduction

*Phaeodactylum tricornutum* Bohlin has been a standard “lab rat” for marine plankton ecologists, cell biologists and physiologists for over a century, despite being a systematic and ecological enigma through much of its history. Bohlin [1] collected and described this alga as a new genus and species from a rock pool. He wrote that the species was triradiate, and believed the fusiform state to be merely one of the Teilungstadien or “cleavage states.” He was uncertain as to the most appropriate higher classification, but suggested that it best fit among diatoms due to its division characteristics and “chemischen Verhalten”, that the chemical behavior of the plastid was that of phycoxanthin, or as it was known to Bohlin, “Diatomin”.

Several useful reviews of experimental analysis of form and shape in *P*. *tricornutum* have been published [2–5]. Here we focus on the taxonomic, nomenclatural and ecological history. We discuss morphology only as it relates to the history of thought as to the ecology of *P*. *tricornutum*.

This diatom was first cultured and studied [6], where it was reported as *Nitzschia closterium* forma *minutissima* Allen and Nelson, but the name was never formerly published and remains a *nomen nudum*. Allen and Nelson [6] noted characteristics which helped make this strain a commonly studied diatom. It remained dispersed and suspended in culture rather than clumping and/or sinking, and the strain showed no tendency towards size reduction (and subsequent size regeneration) in the two years between original isolation and the reported study. They never identified the source of the culture, but Wilson [7] later reviewed Allen’s laboratory notes and found that the now classic strain Plymouth 589 was isolated on agar from plankton from “outside the Sound”, presumably Plymouth Sound. Incidentally, the second classic strain number, Plymouth 569, appears to be an orthographic error on cultures containing the original strain, rather than a separate and unique strain [7].

The first indication that this cultured diatom was not a typical *Nitzschia* (and might be *P*. *tricornutum*) were from observations that a descendant of the original culture contained cells of three distinct shapes: the fusiform or spindle-shaped cells, the triradiate form and the oval form [8]. That paper referred to the diatom simply as *N*. *closterium*, citing Lebour [9] who attributed the authority to Wm. Smith. Barker [8] further observed that the three forms were interconvertible with a gradation of shapes between the three forms. Further he noted that transfers on agar plates resulted in eventual conversion of clones entirely to the oval form, whereas reintroduction into liquid media resulted in rapid conversion to the fusiform shape. The triradiate cells were rare in all conditions. Thus, Barker [8] established that the three forms were environmentally dependent and all part of a single genotype. In retrospect, Barker’s [8] observation that the oval form occurred on agar plates only was the first suggestion that this form might be associated with the benthic habitat. However, Barker himself did not make this connection and his study was in the overall context of marine plankton.

The longevity of *P*. *tricornutum* cultures was made manifest when Wilson [7] noted that the Allen and Nelson (1910) strain was still alive after nearly 40 years, including both the 569 and 589 labelled strains at the Marine Biological Laboratory in Plymouth and at University College in Hull [10]. While he considered *P*. *tricornutum* as a possible identification, he concluded by retaining the name *N*. *closterium* forma *minutissima* and continued to associate this diatom with the plankton environment.

The first published electron microscope images of Plymouth 589 showed fusiform and triradiate cells only, and did not reveal any typical siliceous frustular components [11]. This study concluded that the cultures informally called the “Plymouth *Nitzschia*” were, in fact, *P*. *tricornutum*. Hendy considered, and rejected as unlikely, the possibility that a *Nitzschia* had been originally isolated, but was contaminated and then replaced by *P*. *tricornutum*. Hendey (1954) did not make conclusive comments about its ecology, but again referenced this species in the context of marine plankton.

It was not until 1958 that polymorphic strains still variously identified as *N*. *closterium* forma *minutissima* or *P*. *tricornutum* were recognized as the same species and that this species was unambiguously classified as a diatom [12, 13]. They observed a typical axial area and raphe of a raphid pennate. The margin of the valve was ragged and striae were very fine (as many as 95 in 10 μm). The valves were symmetrical about the transapical axis suggesting a similarity to *Cymbella*. The central area bulged towards what would be called the ventral side in *Cymbella*, sometimes with a pore in the central area on the ventral side. Lewin (1958) created a monotypic family (Phaeodactylaceae) and suborder (Phaeodactylineae) for *P*. *tricornutum*. Lewin et al. (1958) reported chemical differences between oval and fusiform cells, with the former having higher levels of carbohydrates and the latter having higher levels of proteins and lipids per dry weight. Again, in retrospect, this is a potentially significant observation in the context of the life history of *P*. *tricornutum*, and in the context of the adaption and function of diatoms in the plankton and benthic environments.

Johansen [14] was an early formal exploration of the ecological consequences of the multimodal morphology of *Phaeodactylum tricornutum*, hypothesizing that the oval form might be dominant in the small inland Soap Lake because of the preponderance of benthic habitats. Finally, the multimodality of *P*. *tricornutum* morphology has been shown to have inducible planktonic and benthic phases [3] and that several cellular functions and processes are similarly affected by the niche occupied by these forms as suggested by earlier works [13, 15]. In the last several decades, *P*. *tricornutum* has been reported and cultured from a wide variety of locations around the world. These have been characterized genetically and morphologically as the same species [5].

Despite the many observations suggesting a benthic component to this diatom’s life history, it continues to often be discussed explicitly or implicitly as a model diatom in the context of planktonic environments [16, 17–26]. However, several recent papers have recognized the benthic phase as worthy of study. In a review of morphological variation in *P*. *tricornutum*, Martin-Jezequel and Tesson [2] asked an important question: “… why (has) *Phaeodactylum* developed such an adaptation in which a benthic oval form is transformed into two different planktonic forms that seem to exploit the same ecological niche?” The planktonic phase, lacking even a partial valve, would seem to be derived relative to the benthic phase, which retains the plesiomorphic condition of making at least a partial valve made of silica. That is, *P*. *tricornutum* is not a plankton diatom, but a benthic diatom that has evolved morphologically distinct plankton forms.

This interpretation seems probable in that most raphid pennates are benthic, but the phylogenetic position of *P*. *tricornutum* among raphid pennates has not been well-established in formal analyses. Previous multigene molecular phylogenies place *P*. *tricornutum* among the raphids, although placement is variable and not robust even in multigene studies. For example, Theriot et al. [27, 28] included 36 raphid pennates and found two different arrangements of *P*. *tricornutum*, and what they termed “berkeleyoids” and “gomphonemoids” with the same three gene data set and alignment simply by running the data under GTR+G+I and GTR+G models. Theriot et al. [29] with a larger dataset (seven genes, 41 raphid pennates) recovered *P*. *tricornutum* as sister to *Mastogloia* plus the berkeleyoids with bootstrap support of only 21%. Analyzing all protein encoding genes from the chloroplast genome, but only 40 taxa and only seven raphid pennates, Yu et al. [30] recovered *P*. *tricornutum* as sister to *Didymosphenia geminata*. Despite utilizing more than 100 genes, BS support was only 52%.

Here we add more than 250 raphid pennates to a published three gene dataset [31] and report on the phylogenetic position of *P*. *tricornutum*. We recovered *P*. *tricornutum* with 100% bootstrap support as sister to *Gomphonemopsis*, a widely distributed benthic diatom previously classified within the Rhoicospheniaceae in the order Cymbellales [32, 33]. However, neither diatom appears to be closely related to the Cymbellales on the basis of our three gene dataset. While *P*. *tricornutum* exhibits morphological variation that enhances its recruitment into the plankton, we hypothesize that it and many other benthic diatoms are likely to share physiological adaptations which also enhance occasional and temporary recruitment into the plankton.

## Materials and methods

### Taxon sampling and culturing

Collection data for material from which cultures were isolated are provided in S1 File. Material was typically collected by 20 μm mesh plankton net, or by a turkey baster applied directly to the sand and sediment. Strains isolated from the Red Sea were grown in 40 ppt salinity f/2 medium, and maintained at 27°C under a 12:12 hour light:dark photoperiod in a Percival model I-36LL growth chamber. Strains isolated from Guam were also maintained in the Percival model I-36LL growth chamber at 27° C, but in 32 ppt salinity f/2 medium. Other strains were maintained at room temperature (20–24°C) in a north-facing window.

Our *P*. *tricornutum* strains were obtained from the University of Texas Culture Collection of Algae (UTEX). Three strains were grown in f/2 medium (UTEX 642, 646, and 2089) with doubled sodium silicate concentrations, and a fourth (UTEX 640), which had been maintained by UTEX in freshwater soil extract medium, was grown by us in the freshwater COMBO [34] modified by doubling the sodium silicate concentration. All cultures were grown in low light conditions (at the back of a delicatessen type refrigerator with only ambient room fluorescent lighting; light levels not measured) to minimize growth rate with the goal of possibly obtaining maximally silicified valves.

The *Gomphonemopsis* strain utilized (UTKSA0026xx) was initially isolated as an epiphyte on a living *Thalassionema* sp. collected from a subsurface plankton tow in the marina at Duba, Saudi Arabia; unialgal cultures of *Gomphonemopsis* cells were established by re-isolating into fresh culture tubes. We collected 23 samples from the Red Sea coastline in 2015 for floristic analysis to gain a better understanding of the ecology of this *Gomphonemopsis* (S2 File).

### Microscopy preparation

Cultured and wild samples were cleaned in a 1:1:1 mix of 30% hydrogen peroxide and 70% nitric acid. The solution was then washed with distilled water until pH neutral. Cleaned material was then dried onto 22 mm^2^ and 12 mm diameter coverslips for light (LM) and scanning electron microscopy (SEM), respectively. Coverslips for LM were mounted into permanent slides with Naphrax mounting medium. SEM coverslips were mounted on aluminum stubs and coated with 15 nm of iridium using a Cressington 208 Bench Top Sputter Coater. SEM observations were made with a Zeiss SUPRA 40 VP scanning electron microscope at the University of Texas-Austin. Photovouchers for strains are available from the corresponding author.

For whole cell imaging of *P*. *tricornutum* in TEM and SEM, cultured cells were first fixed overnight at room temperature in a mixture of aldehydes (4% glutaraldehyde and 2% formaldehyde in 0.1M cacodylate buffer with 35g/L of Instant Ocean), followed by post-fixation in reduced osmium (2% osmium tetroxide and 2% potassium ferrocyanide in 0.1M cacodylate buffer). Samples for SEM imaging were attached to glass with poly-L-lysine and then dehydrated in ethanol and dried at the critical point of carbon dioxide in a Tousimis Critical Point Dryer (Samdri-790) before sputter coating and imaging as above. Cells for TEM imaging were encased in agar and embedded in epoxy resin (Hard-Plus Resin 812, Electron Microscopy Sciences, Hatfield, PA). Silver to gold sections were collected on Formvar-coated grids and imaged at 80kV with a Tecnai Bio-Twin TEM at the University of Texas-Austin.

### DNA extraction and sequencing

Cultured material was harvested for DNA extraction by pelleting in a Sorvall RC-5B refrigerated superspeed centrifuge (DuPont Company, Newton, CT, USA) for 20 minutes at 7649 x g. Cell pellets were opened by 45 s of cell disruption using a Mini-Beadbeater (Biospec Products, Inc, Bartlesville, OK, USA) with 1.0 mm glass beads. After cell disruption, DNA extraction was done using the QIAGEN DNeasy Plant Mini Kit (QIAGEN Sciences, Valencia, California, USA). Polymerase chain reaction (PCR) amplification and sequencing of small-subunit nuclear rRNA (SSU) and the chloroplast-encoded *rbc*L and *psb*C markers followed the primers and protocols of Ashworth et al. [35].

### Phylogenetic analysis and hypothesis testing

SSU sequences were aligned using SSUalign [36]and a covariance model based on secondary structure models of 33 diatoms [37] as outlined in Li et al [38]. Protein encoding plastid genes (*rbcL* and *psbC*) were aligned using MUSCLE implemented in Seaview 4.0 [39, 40].

SSU data were then partitioned by stem and loop (paired and unpaired sites), and protein encoding data were partitioned by codon position and gene, for a total of eight starting partitions for PartitionFinder 2.0 [41]. As recommended by the documentation, we used the AIC criterion to select the best partitioning scheme: (SSU paired, SSU unpaired, aggregate first codon positions, aggregate second codon positions and aggregate third codon positions).

A maximum likelihood tree was calculated using RAxML v. 8.2.7 [42]. Each dataset was executed under GTR+G for 20 replicates with 500 rapid bootstrap support (BS) replicates. BS values from the run that produced the optimal tree were plotted onto that tree.

Optimal trees were then similarly computed with the exception that *Gomphonemopsis* sp. and *P*. *tricornutum* (together and separately) were constrained to each of several major raphid pennate clades recovered in the optimal run (S3 File). Site likelihood values were calculated for each such optimal constraint tree independently in RAxML. Results were output in TreePuzzle format, and then read into CONSEL where AU tests were performed [43–45].

## Results

### Morphology

#### *Phaeodactylum tricornutum* Bohlin (UTEX640) Figs 1–3

**Fig 1:**
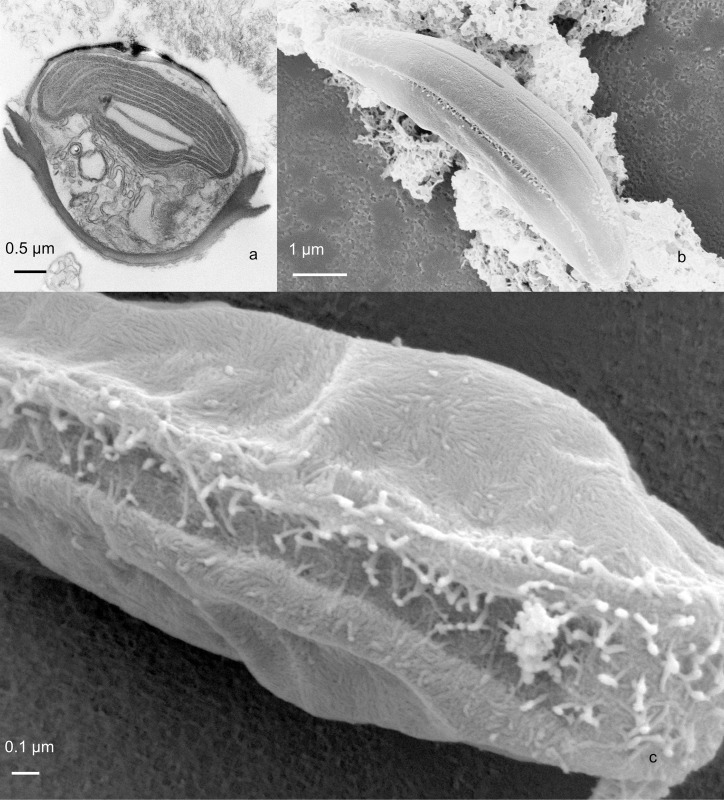
Phaeodactylum tricornutum UTEX640. Whole cells. Fig 1a. TEM thin section of an oval cell across the transapical plane. A single valve is observed at the top of the cell. Fig 1b. SEM of an entire cell after critical point drying, with a visible siliceous valve. Fig. 1c. SEM of a critically point dried cell in girdle view. There was no valve on either side.

**Fig 2:**
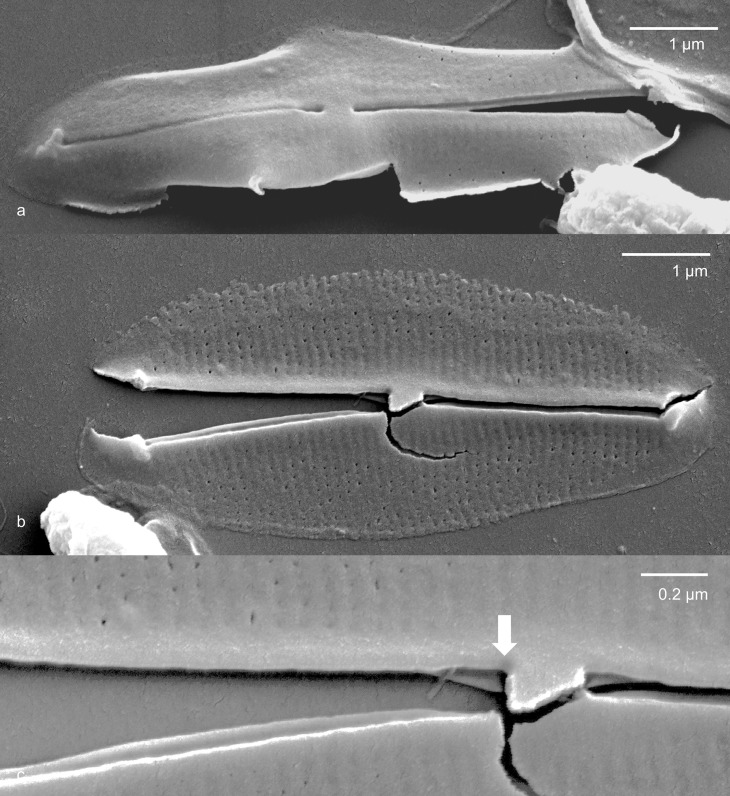
Phaeodactylum tricornutum UTEX640. Valve biological exterior (side towards the environment) from acid cleaned material. Fig 2a. Largely intact valve illustrating simple proximal endings of raphe in exterior view. Fig 2b. Broken valve in external view. 2c. Higher magnification of central area of the specimen in 2b. The arrow indicates the position of the recurved raphe ending, best seen in broken valves or in interior view.

**Fig 3:**
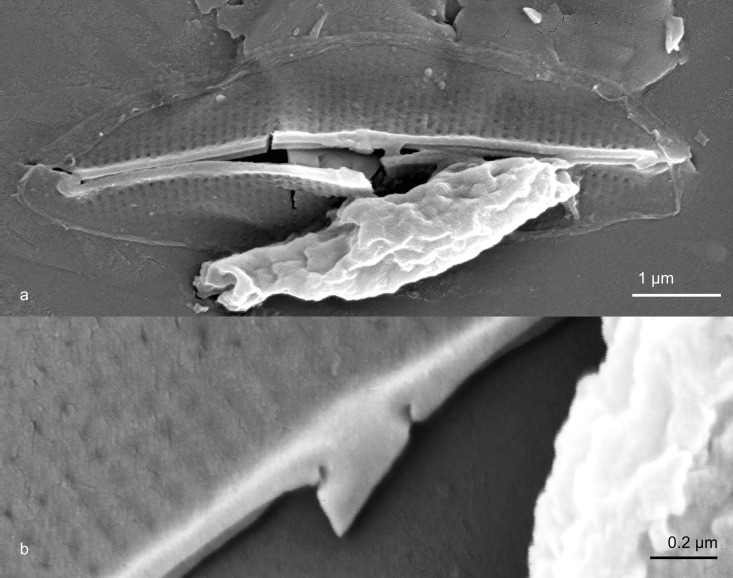
Phaeodactylum tricornutum UTEX640. Valve biological interior (side towards the cell) from acid cleaned material. Fig 3a. Largely intact valve showing greatly thickened longitudinal costae around raphe. Fig. 3b. Central area of a broken valve showing recurved proximal raphe ends.

Only oval cells were observed. Cells forming a valve did so only on one side (Fig 1a and 1b). Many cells did not seem to form valves on either side (Fig 1c). The axial area was relatively heavily silicified in both exterior (Fig 2) and interior (Fig 3) views. Externally, the raphe ended in the central area in a weak expansion (Fig 3a). On the biologically internal surface, the raphe ended was deflected towards one side and then hooked back towards the other side (Figs 2b, 2c and 3b). In interior view, some specimens formed a pair of ridges along the raphe, forming a structure not unlike that of some Berkeleyaceae (Fig 3a). Virgae were dense with 89–92 in 10 μm.

#### *Gomphonemopsis* cf. *exigua* (UTKSA0026xx) Fig 4

**Fig 4:**
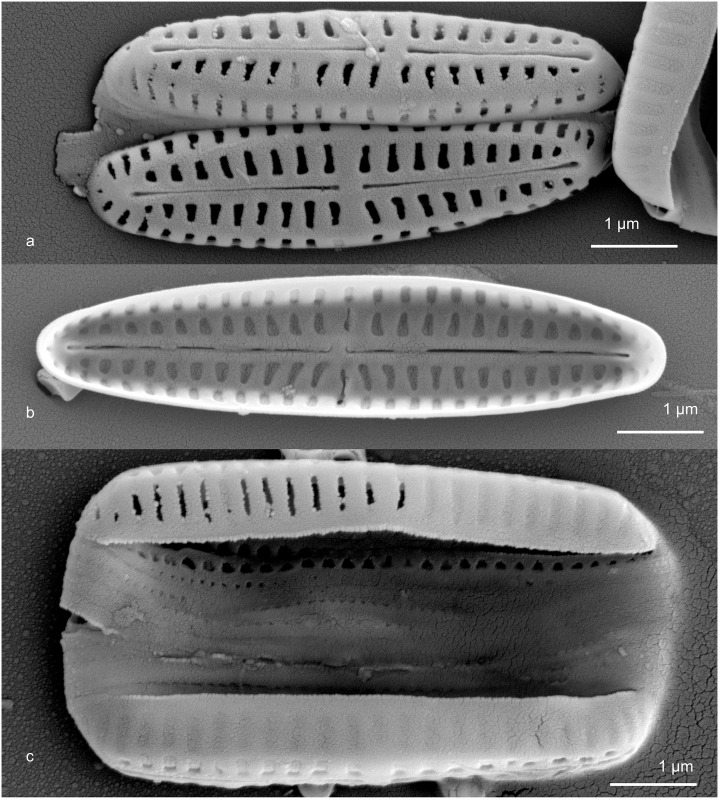
SEMs of *Gomphonemopsis* cf. *exigua* UTKSA0026xx. Fig 4a. Exterior views of both valves of a single specimen of *G*. cf. *exigua*. Fig 4b. Interior view of a valve. Fig. 4c. Girdle view of an entire frustule.

Our specimens were 4–8 μm in length and 0.9–1.5 μm in width, symmetric or nearly so around the apical axis and asymmetric across the transapical axis (Fig 4a and 4b). Transapical virgae were relatively broad. The vimines divide the virgae into two large areolae, their prominence giving the appearance of a costa running the length of the valve at the junction of the face and mantle. The hymenes are delicate looking when observed and appear to have been lost or at least not fully formed on many valves. From the interior, the proximal ends of the raphe slit were deflected towards one side; the distal ends were straight and each terminated in simple helictoglossae. Externally, the proximal and distal ends of the raphe slit were slightly expanded. Copulae were open. The valvocopula had two rows of pores, all other copulae appeared to have one row (Fig 4c). The virgae were variously parallel to the axial area to slightly diverging. The appearance of a distinct central area was thus inconsistent from valve to valve.

#### Distribution of *G*. cf. *exigua* in our Red Sea samples

We recovered *G*.cf. *exigua* in 47% of our samples, although it never occurred in relative abundance higher than 2.8%. In that sample and in most other samples in which the relative abundance of *G*. cf. *exigua* was greater than 1%, the dominant diatom was an unidentified species of *Cocconeis*, a diatom often collected as an epiphyte.

#### Molecular phylogeny and hypothesis testing

**Fig 5:**
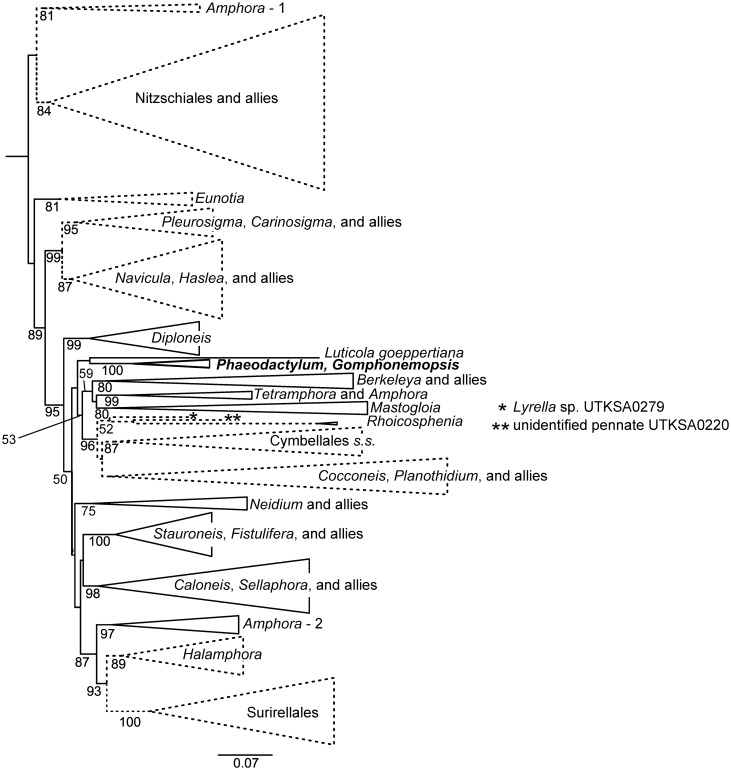
Simplified maximum likelihood tree. The optimal maximum likelihood tree with crown clades collapsed. Numbers at nodes are bootstrap support values of 50% or above. The solid line clades or terminal strains represent taxa which could not be rejected by the AU test as sister to *P*. *tricornutum* plus *G*. cf. *exigua*.

The tree with all individual strains is provided in S1 Fig. A simplified version with crown clades collapsed (Fig 5) has BS supports > 50% indicated; clades that were rejected as sister taxa to *Phaeodactylum* plus *Gomphonemopsis* are indicated with dashes. Overall, the tree had high BS support towards the tips where most crown clades were well supported. However, support for relationships among these groups were uneven. Two nodes towards the base of the tree and two towards the tip received high BS support. The nodes in the middle had very low BS support, generally less than 50%.

All four clones of *P*. *tricornutum* were recovered as a clade with 100% BS support (S1 Fig). *Gomphonemopsis* cf. *exigua* was recovered as sister to *Phaeodactylum* with 100% BS support. This clade, called PG for convenience, was rendered as a single terminal taxon in Fig 5. PG was sister to *Luticola* sp. with low BS support, and that clade was embedded in a larger clade identified as PG and allies. This larger clade included all Cymbellales sequences used in this study. Most Cymbellales formed a monophyletic group (Cymbellales *sensu stricto*), but two *Rhoicosphenia* strains were recovered as sister to an unidentified pennate (UTKSA 0220) and that clade was sister to *Lyrella* sp. Together these taxa form a clade we call Cymbellales *sensu lato* (despite the presence of *Lyrella* sp.). The BS support below this clade was high (96%). Although other nodes between PG and Cymbellales received very low BS support, PG was rejected as a member both of of the Cymbellales *s*.*l*. and *s*.*s* The PG group was also rejected as a member of a clade composed mainly of monoraphid diatoms (labelled “*Cocconeis*, *Planothidium* and allies”).

The nodes between PG and Berkeleyaceae had low BS values, the highest being 59%. Reflecting the weak support for internodes, our data did not reject the possibility that PG was sister to Berkeleyaceae and Amphipleuraceae species. Nor did it reject the possibility of PG being closely related to *Mastogloia* or the clade containing *Tetramphora*.

Other phylogenetically more distant taxa were generally rejected as a close relative of the PG clade (Fig 5). PG was rejected as sister to all crown clades below the node joining *Diploneis* with other diatoms, but a grouping of PG with *Diploneis* itself was not rejected. Relationships among most remaining raphid pennates received low BS support. The only nodes between PG and Allies and other crown clades with high support were among *Amphora*-2, *Halamphora* and Surirellales, and only *Halamphora* and Surirellales were rejected as PG relatives.

## Discussion

Our specimens of *Gomphonemopsis* could not be identified with certainty as any described species of that genus. They were subtly different from *G*. *exigua* (Kütz.) Medlin [32]: the footpoles of our specimens were broadly rather than acutely rounded, the valve faces curved into the mantles rather than being sharply delineated by shape, and the central raphe ends were reflexively curved rather than straight. Rather than describe it as a new species, we elected to identify them as *G*. cf. *exigua*, pending review of type material and within species morphological variation in *Gomphonemopsis* spp.

All *Gomphonemopsis* spp. are reported as diatoms associated with various substrates including artificial substrates, and are often reported as epiphytes on fleshy algae and mangrove roots [32, 46–53]. Given the close relationship of *P*. *tricornutum* to *Gomphonemopsis*, it seems a safe assumption that *P*. *tricornutum*, or the lineage that immediately gave rise to it, was a benthic diatom and that the morphotypes associated with the plankton environment are a later adaptation. While this conclusion is not a huge surprise, given that the benthic habitat appears to be predominate for raphid pennates [54], we note that the literature on *P*. *tricornutum* often still explicitly or implicitly treats it as a model for plankton diatoms [16, 17–26] rather than as a model for benthic diatoms. The potential broader significance of this has yet to be investigated.

One might ask whether it matters if a model diatom is benthic or planktonic. We argue that this is a question worth investigating in more detail. *Phaeodactylum* is unusual in that it is the only diatom known to have specific polymorphic changes that appear to aid in its recruitment from the benthos to the plankton and vice versa [2, 55]. There are several correlated changes in physiology and biochemistry. Environmental conditions and age of culture trigger changes in polysaccharide concentrations, which lead to aggregation and sinking [3, 5]. Cell biologists have identified biochemical differences between the fusiform/spindle-shaped *P*. *tricornutum* and the oval form [3, 5, 15].

Many benthic diatoms are found in plankton samples but they are typically regarded as passively entrained into the plankton environment, being called tychoplankton (“tycho-”from the Greek τυχαίος, which transliterates to “tychaìos”, meaning random, casual or accidental). Based on observations cited above it appears that *P*. *tricornutum* is not an accidental tourist in the plankton environment, but has evolved a multi-modal morphology as an adaptation to incursions into the plankton. Might the physiological changes noted in those papers also represent evolved traits promoting active recruitment into the plankton?

Tychoplanktonic diatoms can contribute a majority component to planktonic primary productivity [56], suggesting that they remain physiologically active in the plankton. It would not be surprising then that, given differential nutrient availability and grazing pressure associated with different plankton and benthic habitats throughout the year, other diatoms may also have developed adaptations associated with active recruitment into the plankton. At the very least recruitment of benthic diatoms into the plankton is important in their dispersal [57], which can be important in their evolutionary success.

Changes in the degree of silicification of the valve is one possible phenotypic alteration that might occur in diatoms related to planktonic vs. benthic habitat. In fact, it has been speculated that having a silicified valve also enhances the benthic nature of *P*. *tricornutum* [2]. Indeed, marine benthic araphid diatoms appear to be more heavily structured than their planktonic relatives [58], but no comparative measures of actual silica content have been made in a phylogenetic context. Conley et al. [59] found that marine benthic raphid diatoms do contain more silica per unit biovolume than planktonic raphids. However, there is no difference in silica content between freshwater planktonic diatoms and freshwater benthic diatoms [59]. This provides one challenge to the idea that differences in silica content are a necessary difference between plankton and benthic diatoms. Perhaps the difference in silica content is not related to sinking, but is related to different degrees of silica availability versus demand in the marine benthic and planktonic environments, the same way silica availability might explain difference in silicification in marine and freshwater plankton [60].

It is known that buoyancy is affected by factors other than silica content, and that the differences between benthic and planktonic diatoms are not merely based on buoyancy. The extracellular threads of planktonic Thalassiosirales increase form resistance to sinking [61]. Aggregation of diatoms due to stickiness of extracellular polysaccharides may also affect sinking [62] and this is certainly true for *P*. *tricornutum* where several studies have shown the benthic, oval form tended to aggregate under environmental conditions and that this is associated with changes in extracellular polysaccharide composition [2, 3, 5]. Protoplast solute content affects buoyancy [63], and changes in solute content can occur more rapidly (over hours or days) within an existing cell, whereas silicification changes are moderated mainly at cell division. These factors all contribute to change in buoyancy in *P*. *tricornutum* [2].

Buoyancy may be affected by carbohydrate concentration, which in turn, may be affected by nutrient status in several algae in the marine and freshwater environments. *Microcystis* buoyancy can be regulated by light and nutrients. Excess photosynthetic energy may be stored as carbohydrate ballast, which can cause cells to sink until respiration has reduced that balance and the colonies become buoyant and able to outcompete non-buoyant algae again [64–67]. As a result, benthic cyanobacteria recruited into the plankton can constitute a major source of phosphorous (P) loading in lakes, and some even appear to undergo luxury consumption of P as part of the benthos [68].

Regulation of buoyancy by carbohydrate ballasting occurs in the planktonic diatom *Rhizosolenia*. Shifts of *Rhizosolenia* mats from deep NO_3_^-^ rich layers into the photic zone of the open ocean are an apparent life history adaptation [69–71]. The behavior of these mats is an important component of N-loading to the photic zone [70]. The metabolic mechanism is that the accumulation of carbohydrates under photosynthesis causes the diatom to become negatively buoyant, whereupon it sinks, and the carbohydrate is metabolized in the reduction of NO_3_^-^.

Khodse and Bhosle [72] studied *Amphora rostrata* Wm. Smith and provided the most direct evidence that some benthic diatoms may be genetically programmed to release from substrates as an adaptive strategy. They observed that cultures of this species were composed of a combination of cells attached to the flask wall and unassociated cells. They found that attached cells had higher concentrations of glucose and glucosamine, and lower concentrations of fucose. They also observed that cells in stationary phase contained more fucose and less glucose, consistent with the observations of Percival [73]. Incidentally, Roszkowski et al. [74] noted that fucose rich polysaccharide inhibited the adhesion of certain cancer cells in mice, and then suggested that fucose rich carbohydrates might also prevent diatom adhesion to the culture flask surface. To our knowledge, this hypothesis, while highly speculative, has not yet been tested.

The significance of fucose content is worth further study. It has been observed to increase with age or growth or estuarine conditions in some diatoms [75, 76], and glucose rich polysaccharides were replaced by fucose rich polysaccharides during the stationary phase of growth of some diatoms [73]. Perhaps fucose, if not being directly relevant, is an indicator of general carbohydrate excess in nutrient stressed diatoms, and are only reflective of carbohydrates involved in ballast. And, of course, diatom buoyancy is regulated by factors other than carbohydrate content. Regardless of the role of fucose, the results of Khodse and Bhosle [72] strongly suggest that *P*. *tricornutum* is not the only “benthic” diatom that may have evolved life history and metabolic strategies that aid in their occasional purposeful rather than stochastic recruitment to the plankton.

It may be that *P*. *tricornutum* retained the plesiomorphic oval shape along with the modicum of silicification for reasons entirely other than buoyancy. The oval form of *P*. *tricornutum* is motile and this motility is attributed to the possession of a raphe [12, 13, 77]. The loss of a raphe would likely be of little consequence to the planktonic form, but of great significance to the benthic form for both initial attachment and motility [78]. Thus, *P*. *tricornutum* may be under selection to retain only as much of the valve as is necessary to make a functional raphe.

In summary, our phylogenetic results indicate that *P*. *tricornutum* is closely related to the small, weakly asymmetric benthic diatom *Gomphonemopsis*. The next closest relative of this group from a morphological standpoint would seem to be Cymbellaceae, but the conflict with this classification is statistically significant. Inclusion of other benthic raphid pennates is likely necessary to better resolve the position of *P*. *tricornutum* (and *Gomphonemopsis*). Our recent study using the entire chloroplast genome [26] lacks *Gomphonemopsis* and links *P*. *tricornutum* to the only Cymbellaceae exemplar in the dataset (*Didymosphenia geminata* [Lyngbye] M. Schmidt) but with only 52% BS support. Thus, while *P*. *tricornutum* remains a model diatom because of the extraordinary amount of genomic and metabolomic work performed on it, its relevance to the plankton environment *per se* is unclear at best.

## Supporting information

S1 Fig Complete topology of ML phylogenetic tree of pennate diatoms in our three gene (nuclear SSU, *rbcL*, *psbC*) dataset. Terminal taxa are identified by name and strain number. Numbers associated with nodes are bootstrap support values.
(TIF)

S1 File Strain identification and Genbank sequence numbers for newly accessioned material. (DOC)

S2 File Localities for diatom floristic survey to determine distribution of *Gomphonemopsis* cf. *exigua*. (XLSX)

S3 File Constraint trees used in hypothesis testing. All trees are in Newick format.
(TXT)
