# Supplementary material for: Phylogenetic analysis and a review of the history of the accidental phytoplankter, Phaeodactylum tricornutum Bohlin (Bacillariophyta)
Source: PLoS One. 2018 Jun 8;13(6):e0196744. doi: 10.1371/journal.pone.0196744 (PMC5993285; doi:10.1371/journal.pone.0196744)
Supplement: S1 File — (DOC) [file pone.0196744.s002.doc]

Table S1. Taxa and DNA sequence data used in phylogenetic analysis, with strain voucher ID and GenBank accession numbers. Collection site for original sample is also included (where known); in the case of cultures from public collections, the culture ID is provided here (UTEX = UTEX Culture Collection of Algae; NCMA = National Center for Marine Algae and Microbiota; CSIRO = Australian National Algae Culture Collection; MCC-NIES = Microbial Culture Collection at National Institute for Environmental Studies). Raphid pennate taxa (ingroup) provided first in the table; araphid pennate taxa (outgroup) follow after table break. Taxa are listed alphabetically.

| Taxon | Strain Voucher | Collection Site  (Locality in paretheses) | GenBank Accession  (SSU, *rbc*L, *psb*C) |
| --- | --- | --- | --- |
| *Achnanthes chlidanos* M.H.Hohn & Hellerman |  |  | KJ658412, KJ658394, N/A |
| *Achnanthes coarctata* (Brébisson ex W. Smith) Grunow in Cleve & Grunow | HK079 | FD185 (UTEX) | HQ912594, HQ912458, HQ912287 |
| *Achnanthes sp* Bory | HK303 | SanNicholas1 (San Nicholas, Canary Islands) | KC309473, KC309545, KC309617 |
| *Achnanthes sp* Bory | HK309 | ECT3883 (Rainbow Harbor, Long Beach, California) | KC309474, KC309546, KC309618 |
| *Achnanthes sp* Bory | HK310 | ECT3911 (Long Beach, California) | KC309475, KC309547, KC309619 |
| *Achnanthes sp* Bory | HK311 | ECT3684 (Achang Reef, Guam) | KC309476, KC309548, KC309620 |
| *Achnanthes sp* Bory | HK517 | Azo42 (Azores) | MH063437, MH064054, MH063967 |
| *Achnanthes sp* Bory | UTKSA0263 | KSA2015-16 (Al-Nawras, Jeddah, Saudi Arabia) | MH063438, MH064055, N/A |
| *Achnanthidium minutissimum* (Kützing) Czarnecki |  |  | AM502032, AM710499, N/A |
| *Adlafia brockmannii* (Hustedt) Bruder & Hinz |  |  | AM502020, AM710487, N/A |
| *Amphipleura pellucida* Kützing | HK287 | ECT3568 (Lake Travis, Texas) | KC309477, KC309549, KC309621 |
| *Amphora aliformis* | AMPH177 |  | KP229525, KP229546, KP229548 |
| *Amphora caribaea* | AMPH086 |  | KJ463428, KJ463458, KJ463488 |
| *Amphora commutata* | AMPH126 |  | KP229526, KP229547, KP229549 |
| *Amphora cf immarginata* Nagumo | UTKSA0172 | KSA2015-37 (Rabigh, Saudi Arabia) | MH063439, MH064056, MH063968 |
| *Amphora helenensis* | SZCZCH704 |  | KT943649, KT943672, KT943709 |
| *Amphora cf helenensis* | SZCZP12 |  | KU179126, KU179113, KU179140 |
| *Amphora hyalina* | AMPH136 |  | KJ463432, KJ463462, KJ463492 |
| *Amphora lineolata* | AMPH035 |  | KJ463435, KJ463465, KJ463495 |
| *Amphora obtusa* Gregory | UTKSA0275 | KSA2015-37 (Rabigh, Saudi Arabia) | MH063440, MH064057, N/A |
| *Amphora obtusa v crassa* | AMPH070 |  | KJ463436, KJ463466, KJ463496 |
| *Amphora pediculus* (Kützing) Grunow |  | L1030 (UTEX) | HQ912417, HQ912403, HQ912389 |
| *Amphora securicula* | AMPH046 |  | KJ463440, KJ463470, KJ463500 |
| *Amphora sp.* Ehrenberg ex Kützing | HK502 | PackaryChannelSediment (Mustang Island, Texas) | MH017634, MH064058, MH063969 |
| *Amphora sp.* Ehrenberg ex Kützing | UTKSA0087 | SA12 (Markaz Al Shoaibah, Saudi Arabia) | MH063441, MH064059, MH063970 |
| *Amphora sp.* Ehrenberg ex Kützing | UTKSA0115 | KSA2015-27 (Markaz Al Shoaibah, Saudi Arabia) | MH063442, MH064060, N/A |
| *Amphora sp.* Ehrenberg ex Kützing | UTKSA0153 | KSA2015-37 (Rabigh, Saudi Arabia) | MH063443, MH064061, MH063971 |
| *Amphora sp.* Ehrenberg ex Kützing | UTKSA0177 | KSA2015-41 (Rabigh, Saudi Arabia) | MH063444, MH064062, MH063972 |
| *Amphora sublaevis* | AMPH135 |  | KJ463444, KJ463474, KJ463504 |
| *Amphora subtropica* | AMPH051 |  | KJ463445, KJ463475, KJ463505 |
| *Amphora sulcata* | AMPH083 |  | KJ463446, KJ463476, KJ463506 |
| *Amphora vixvisibilis* Li & Witkowski | SZCZCH967 |  | KT943648, KT943670, KT943706 |
| *Amphora waldeniana* | AMPH011 |  | KJ463447, KJ463477, KJ463507 |
| *Anomoeoneis fogedii* Reimer |  | FD399 (UTEX) | KJ011610, KJ011793, N/A |
| *Anomoeoneis sphaerophora* Pfitzer |  | FD160 (UTEX) | KJ011612, KJ011795, N/A |
| *Astartiella sp.* A.Witkowski, Lange-Bertalot & Metzeltin | UTKSA0146 | KSA2015-11 (Bhadur Resort, Saudi Arabia) | MH063445, MH064063, MH063973 |
| *Astartiella sp.* A.Witkowski, Lange-Bertalot & Metzeltin | SZCZCH151 |  | N/A, KT943613, KT943624 |
| *Auricula sp.* Castracane | HK434 | 21IV14-4D (Rabbit Key Basin, Florida) | KX981842, KX981810, KX981789 |
| *Auricula cf complexa* (Gregory) Cleve | UTKSA0038 | SA12 (Markaz Al Shoaibah, Saudi Arabia) | MH063446, MH064064, MH063974 |
| *Auricula cf flabelliformis* M. Voigt | UTKSA0071 | SA12 (Markaz Al Shoaibah, Saudi Arabia) | MH063447, MH064065, MH063975 |
| *Bacillaria paxillifer* (O. F. Müller) T. Marsson | HK130 | FD468 (UTEX) | HQ912627, HQ912491, HQ912320 |
| *Bacillaria sp.* J.F.Gmelin | HK475 | GU44BK-1 (Gab Gab Beach, Guam) | MH063448, MH064066, MH063976 |
| *Bacillaria sp.* J.F.Gmelin | UTKSA0009 | SA27 (Jeddah, Saudi Arabia) | MH063449, MH064067, MH063977 |
| *Bacillaria sp.* J.F.Gmelin | UTKSA0129 | KSA2015-9 (Bhadur Resort, Saudi Arabia) | MH063450, MH064068, MH063978 |
| *Bacillaria sp.* J.F.Gmelin | UTKSA0130 | KSA2015-9 (Bhadur Resort, Saudi Arabia) | MH063451, MH064069, MH063979 |
| *Berkeleya hyalina* (F.E.Round & M.E.Brooks) E.J.Cox | HK388 | ECT3614 (La Jolla, California) | KJ577847, KJ577882, KJ577917 |
| *Berkeleya rutilans* (Trentepohl ex Roth) Grunow | HK154 | ECT3616 (Laguna Beach, California) | HQ912637, HQ912501, HQ912330 |
| *Berkeleya rutilans* (Trentepohl ex Roth) Grunow | HK389 | ECT3602 (Bolinas, California) | KJ577848, KJ577883, KJ577918 |
| *Biremis sp.* D.G. Mann & E.J. Cox | HK438 | 21IV14-2A (Duck Key, Florida) | KX981835, KX981811, N/A |
| *Biremis sp.* D.G. Mann & E.J. Cox | UTKSA0352 | KSA2016-19 (Rabigh, Saudi Arabia) | MH063452, MH064070, MH063980 |
| *Caloneis lewisii* Patrick | HK060 | FD54 (UTEX) | HQ912580, HQ912444, HQ912273 |
| *Caloneis sp.* P.T. Cleve | KSA0127 | SA12 (Markaz Al Shoaibah, Saudi Arabia) | KU179135, KU179125, N/A |
| *Caloneis sp.* P.T. Cleve | HK429 | SantaRosa cor.green (Costa Rica) | KU179134, KU179123, N/A |
| *Caloneis cf linearis* (Cleve) Boyer | HK430 | 21IV14-3A (Captain’s Key, Florida) | KU179132, KU179119, KU179146 |
| *Caloneis cf excentrica* (Grunow) Boyer | HK431 | 21IV14-2A (Duck Key, Florida) | KU179130, KU179117, KU179144 |
| *Caloneis sp.* P.T. Cleve | HK477 | GU7Y-4 (University of Guam Marine Laboratories, Guam) | MH063453, MH064071, MH063981 |
| *Caloneis sp.* P.T. Cleve | HK479 | GU52V-2 (Outhouse Beach, Guam) | N/A, MH064072, MH063982 |
| *Caloneis sp.* P.T. Cleve | UTKSA0235 | KSA2015-37 (Rabigh, Saudi Arabia) | MH063454, MH064073, MH063983 |
| *Caloneis sp.* P.T. Cleve | UTKSA0252 | KSA2015-42 (Rabigh, Saudi Arabia) | MH063455, MH064074, MH063984 |
| *Caloneis cf westii* (W. Smith) Hendey | SZCZCH1002 |  | KT943628, KT943654, KT943687 |
| *Campylodiscus clypeus* (Ehrenberg) Kützing |  | L951 (UTEX) | HQ912412, HQ912398, HQ912384 |
| *Campylodiscus sp.* Ehrenberg ex Kützing |  | ECT3613 (Tomales Bay, California) | HQ912413, HQ912399, HQ912385 |
| *Campylodiscus sp.* Ehrenberg ex Kützing | UTKSA0284 | KSA2015-29 (Markaz Al Shoaibah, Saudi Arabia) | MH063456, MH064075, N/A |
| *Carinasigma minuta* (Donkin) G. Reid | HK418 | GU7X-6 (University of Guam Marine Lab, Guam) | KX981841, KX981812, KX981790 |
| *Climaconeis riddleae* Prasad | HK178 | ECT3724 (Umatac Bay, Guam) | HQ912644, HQ912508, HQ912337 |
| *Climaconeis sp.* Grunow | UTKSA0040 | SA26 (Jeddah, Saudi Arabia) | KX981836, KX981813, N/A |
| *Climaconeis undulata* (Meister) Lobban et al | HK218 | ECT3743 (Talofofo Bay, Guam) | KC309478, KC309550, N/A |
| *Cocconeis cf cupulifera* Riaux-Gobin, Romero, Compère & Al-Handal | SZCZCH662 |  | N/A, KT943680, KT943718 |
| *Cocconeis cf mascarenica* Riaux-Gobin & Compère | SZCZCH283 |  | N/A, KT943679, KT943717 |
| *Cocconeis placentula* Ehrenberg | HK077 | FD23 (UTEX) | HQ912592, HQ912456, HQ912285 |
| *Cocconeis stauroneiformis* (W. Smith) H. Okuna | s0230 |  | AB430614, AB430694, N/A |
| *Cocconeis sp* Ehrenberg | UTKSA0056 | SA28 (Jeddah, Saudi Arabia) | KU179133, KU179120, KU179147 |
| *Cocconeis sp* Ehrenberg | HK312 | ECT3901 (Channel #5, US-1, Florida) | KC309479, KC309551, KC309622 |
| *Cocconeis sp* Ehrenberg | SZCZP67 |  | KT943600, KT943614, KT943625 |
| *Craspedostauros alyoubii* J. Sabir & Ashworth | UTKSA0083 | SA18 (Duba, Saudi Arabia) | KX981857, KX981814, KX981791 |
| *Craspedostauros amphoroides* (Grunow) Cox | HK447 | CCMP797 (NCMA) | KX981859, KX981815, N/A |
| *Craspedostauros paradoxa* Ashworth & Lobban | HK441 | GU44BK-1 (Gab Gab Beach, Guam, USA) | KX981858, KX981816, KX981792 |
| *Craspedostauros* cf *neoconstrictans* Cox | HK448 | CCMP1120 (NCMA) | KX981860, KX981817, KX981793 |
| *Craticula cuspidata* (Kützing) Mann | HK061 | FD35 (UTEX) | HQ912581, HQ912445, HQ912274 |
| *Cylindrotheca closterium* (Ehrenberg) Reimann & Lewin | HK180 | CCMP1855 (NCMA) | HQ912645, HQ912509, HQ912338 |
| *Cylindrotheca sp.*  Rabenhorst | UTKSA0079 | SA12 (Markaz Al Shoaibah, Saudi Arabia) | KX981848, KX981826, KX981801 |
| *Cylindrotheca sp.*  Rabenhorst | UTKSA0082 | SA18 (Duba, Saudi Arabia) | KX981847, KX981827, KX981802 |
| *Cymatoneis sp.* Cleve | UTKSA0378 | KSA2016-3 (Bhadur Resort, Saudi Arabia) | MH063457, MH064076, MH063985 |
| *Cymatopleura elliptica* (Brebisson ex Kutzing) W. Smith | HK215 | L1333 (UTEX) | HQ912659, HQ912523, HQ912352 |
| *Cymbella aspera* (Ehrenberg) Cleve |  | FD272 (UTEX) | KJ011615, KJ011797, N/A |
| *Cymbella proxima* Reimer |  |  | AM502017, AM710484, N/A |
| *Cymbopleura naviculiformis* (Auerswald ex Heiberg) Krammer |  |  | AM502004, AM710471, N/A |
| *Denticula kuetzingii* Grunow | HK104 | FD135 (UTEX) | HQ912610, HQ912474, HQ912303 |
| *Didymosphenia geminata* (Lyngbye) M. Schmidt | CH058 |  | KJ011636, KJ011819, N/A |
| *Diploneis cf cheronensis* (Grunow) Cleve | HK417 | GU44AY-6 (Gab Gab Beach, Guam) | MH017637, MH064077, MH063986 |
| *Diploneis* cf *smithii* (Brébisson in W. Smith) P.T. Cleve | HK437 | GU44AY-6 (Gab Gab Beach, Guam) | KX981837, KX981818, KX981794 |
| *Diploneis parca* (Schmidt in Schmidt et al.) Boyer | UTKSA0267 | KSA2015-49 (Duba, Saudi Arabia) | MH063458, MH064078, MH063987 |
| *Diploneis cf smithii* (Brébisson in W. Smith) P.T. Cleve | UTKSA0232 | KSA0215-30 (Markaz Al Shoaibah, Saudi Arabia) | MH063459, MH064079, N/A |
| *Diploneis cf smithii* (Brébisson in W. Smith) P.T. Cleve | UTKSA0238 | KSA2015-37 (Rabigh, Saudi Arabia) | MH063460, MH064080, MH063988 |
| *Diploneis sp.* (Ehrenberg) P.T. Cleve | HK435 | Coz-4 (Cozumel, Mexico) | KX981839, KX981819, KX981795 |
| *Diploneis sp.* (Ehrenberg) P.T. Cleve | HK436 | Coz-4 (Cozumel, Mexico) | KX981838, KX981820, KX981796 |
| *Diploneis sp.* (Ehrenberg) P.T. Cleve | HK483 | 21IV14-2A (Duck Key, Florida) | MH017638, MH064081, MH063989 |
| *Diploneis sp.* (Ehrenberg) P.T. Cleve | HK484 | PackaryChannelPlank (Mustang Island, Texas) | MH017639, MH064082, MH063990 |
| *Diploneis sp.* (Ehrenberg) P.T. Cleve | UTKSA0190 | KSA2015-14 (Bhadur Resort, Saudi Arabia) | MH063461, MH064083, MH063991 |
| *Diploneis subovalis* Cleve | HK084 | FD282 (UTEX) | HQ912597, HQ912461, HQ912290 |
| *Diploneis vacillans* (A.W.F. Schmidt) Cleve | UTKSA0145 | KSA2015-11 (Bhadur Resort, Saudi Arabia) | MH063462, MH064084, MH063992 |
| *Diploneis vacillans* (A.W.F. Schmidt) Cleve | UTKSA0150 | KSA2015-37 (Rabigh, Saudi Arabia) | N/A, MH064085, MH063993 |
| *Diploneis vacillans* (A.W.F. Schmidt) Cleve | UTKSA0221 | KSA2015-7 (Bhadur Resort, Saudi Arabia) | N/A, MH064086, MH063994 |
| *Donkinia sp.* Ralfs | UTKSA0269 | KSA2015-37 (Rabigh, Saudi Arabia) | MH063463, MH064087, MH063995 |
| *Encyonema norvegica* (Grunow) Mayer |  | FD342 (UTEX) | KJ011643, KJ011826, N/A |
| *Entomoneis ornata* (Bailey) Reimer |  |  | HQ912411, HQ912397, HQ912383 |
| *Entomoneis sp.* Ehrenberg | HK 135 | CS782 (CSIRO) | HQ912631, HQ912495, HQ912324 |
| *Entomoneis sp.* Ehrenberg | SZCZM496 |  | KT943630, KT943656, KT943689 |
| *Entomoneis sp.* Ehrenberg | UTKSA0013 | SA12 (Markaz Al Shoaibah, Saudi Arabia) | N/A, MH064088, MH063996 |
| *Entomoneis sp.* Ehrenberg | UTKSA0061 | SA18 (Duba, Saudi Arabia) | MH063464, MH064089, MH063997 |
| *Entomoneis sp.* Ehrenberg | UTKSA0080 | SA12 (Markaz Al Shoaibah, Saudi Arabia) | MH063465, MH064090, MH063998 |
| *Entomoneis sp.* Ehrenberg | UTKSA0092 | SA18 (Duba, Saudi Arabia) | MH063466, MH064091, MH063999 |
| *Eolimna minima* (Grunow in Van Heurck) H. Lange-Bertalot |  |  | AM501962, AM710427, N/A |
| *Epithemia argus* (Ehrenberg) Kützing | CH211 |  | HQ912408, HQ912394, HQ912380 |
| *Epithemia sorex*  Kützing | CH148 |  | HQ912409, HQ912395, HQ912381 |
| *Eunotia curvata* Lagerstedt | HK086 | FD412 (UTEX) | HQ912599, HQ912463, HQ912292 |
| *Eunotia glacialis* Meister | HK069 | FD46 (UTEX) | HQ912586, HQ912450, HQ912279 |
| *Eunotia pectinalis* (Kützing) Rabenhorst | HK153 | NIES461 (MCC-NIES) | HQ912636, HQ912500, HQ912329 |
| *Eunotia sp.* Ehrenberg | HK286 | ECT3676 (Tinago River, Guam) | KC309480, KC309552, KC309623 |
| *Fallacia monoculata* (Hustedt) Mann | HK082 | FD254 (UTEX) | HQ912596, HQ912460, HQ912289 |
| *Fallacia pygmaea* (Kützing) Stickle & Mann | HK093 | FD294 (UTEX) | HQ912605, HQ912469, HQ912298 |
| *Fallacia sp.* Stickle & D.G. Mann | HK482 | GU52X-3 (Outhouse Beach, Guam) | MH063467, MH064092, MH064000 |
| *Fistulifera pelliculosa* (Brebisson) Lange-Bertalot |  |  | AY485454, HQ337547, N/A |
| *Fistulifera saprophila* (Lange-Bertalot & Bonik) Lange-Bertalot |  |  | KC736618, KC736593, N/A |
| *Geissleria decussis* (Østrup) Lange-Bertalot & Metzeltin |  | FD050 (UTEX) | KJ011647, KJ011830, N/A |
| *Gomphonema affine* Kützing | HK098 | FD173 (UTEX) | HQ912608, HQ912472, HQ912301 |
| *Gomphonema parvulum* (Kützing) Kützing | HK081 | FD241 (UTEX) | HQ912595, HQ912459, HQ912288 |
| *Gomphonemopsis cf pseudoexigua* (Simonsen) Medlin | UTKSA0026x | SA18 (Duba, Saudi Arabia) | MH063471, MH064098, MH064005 |
| *Gyrosigma acuminatum* (Kützing) Rabenhorst | HK085 | FD317 (UTEX) | HQ912598, HQ912462, HQ912291 |
| *Halamphora catenulafalsa* Witkowski & Ch. Li | SZCZCH452 |  | KT943646, KT943669, KT943704 |
| *Halamphora coffeaeformis* (Agardh) Levkov | HK089 | FD75 (UTEX) | HQ912602, HQ912466, HQ912295 |
| *Halamphora cf costata* (Smith) Levkov | UTKSA0195 | KSA2015-22 (Markaz Al Shoaibah, Saudi Arabia) | MH063468, MH064093, MH064001 |
| *Halamphora coloradiana* J.G. Stepanek & J.P. Kociolek | AMPH025 |  | KJ463450, KJ463480, KJ463510 |
| *Halamphora montana* (Krasske) Levkov | TCC477 |  | KC736615, KC736590, N/A |
| *Halamphora normanii* (Rabenhorst) Levkov |  |  | AM501958, AM710424, N/A |
| *Halamphora oligotraphenta* (Lange-Bertalot) Levkov | AMPH009 |  | KJ463451, KJ463481, KJ463511 |
| *Halamphora sp.* (Cleve) Levkov | SZCZCH101 |  | KT943645, KT943682, KT943703 |
| *Halamphora sp.* (Cleve) Levkov | SZCZCH623 |  | KT943647, KT943684, KT943705 |
| *Halamphora sp.* (Cleve) Levkov | SZCZCH975 |  | KT943650, KT943673, KT943710 |
| *Halamphora veneta* (Kützing) Levkov | AMPH005 |  | KJ463452, KJ463482, KJ463512 |
| *Hantzschia amphioxys v. major* Grunow in Van Heurck |  |  | HQ912404, HQ912390, HQ912376 |
| *Haslea cf howeana* (Hagelstein) Giffen | HK494 | GU7Y-4 (University of Guam Marine Labs, Guam) | N/A, MH040268, MH040241 |
| *Haslea cf howeana* (Hagelstein) Giffen | HK496 | PR6 (San Juan, Puerto Rico) | MH017640, MH040269, MH040242 |
| *Haslea cf howeana* (Hagelstein) Giffen | UTKSA0211 | KSA2015-54 (Duba, Saudi Arabia) | MH063469, MH064094, MH064002 |
| *Haslea ostrearia* (Gaillon) Simonsen | NCC158.4 |  | N/A, HF563525, HF558667 |
| *Haslea ostrearia* (Gaillon) Simonsen | NCC321 |  | N/A, HF563527, HF558669 |
| *Haslea sp.* Simonsen | KSA0102 | SA4 (Durrah, Saudi Arabia) | KX981844, KX981821, KX981797 |
| *Haslea sp.* Simonsen | HK493 | GU52X-1 (Outhouse Beach, Guam) | N/A, MH064095, MH064003 |
| *Haslea sp.* Simonsen | UTKSA0122 | KSA2015-30 (Markaz Al Shoaibah, Saudi Arabia) | N/A, MH064096, MH064004 |
| *Hippodonta capitata* (Ehrenberg) Lange-Bertalot, Metzeltin & Witkowski |  |  | AM501966, AM710432, N/A |
| *Hydrosilicon mitra* Brun | UTKSA0421 | KSA2015-37 (Rabigh, Saudi Arabia) | MH063470, MH064097, N/A |
| *Lemnicola hungarica* (Grunow) Round | HK129 | FD456 (UTEX) | HQ912626, HQ912490, HQ912319 |
| *Luticola goeppertiana* (Bleisch) D.G.Mann ex J.Rarick, S.Wu, S.S.Lee & Edlund |  |  | AM501967, AM710433, N/A |
| *Lyrella hennedyi* (W. Smith) Stickle & Mann | UTKSA0279 | KSA2015-5 (Bhadur Resort, Saudi Arabia) | MH063472, MH064099, |
| *Mastogloia aquilegiae* Grunow in Moller | UTKSA0224 | KSA2015-49 (Duba, Saudi Arabia) | N/A, MH064100, MH064007 |
| *Mastogloia fimbriata* (T. Brightwell) Grunow | HK485 | GU52X-1 (Outhouse Beach, Guam) | MH040321, MH040270, MH040243 |
| *Mastogloia cf pumila* (Grunow) Cleve | HK136 | 29X07-6B (Mustang Island, Texas) | HQ912632, HQ912496, HQ912325 |
| *Mastogloia sp.* Thwaites in W. Smith | HK314 | ECT3762 (Taeleyag Beach, Guam) | KC309481, KC309553, N/A |
| *Mastogloia sp.* Thwaites in W. Smith | KSA0062 | SA17 (Duba, Saudi Arabia) | MH063473, MH064101, MH064008 |
| *Mastogloia sp.* Thwaites in W. Smith | UTKSA0313 | KSA0216-44 (Markaz Al Shoaibah, Saudi Arabia) | MH063474, MH064102, MH064009 |
| *Mayamea perimitis* (Hustedt) K. Bruder & L.K. Medlin | TCC540 |  | KC736630, KC736600, N/A |
| *Meuniera membranacea* (Cleve) P. C. Silva | HK313 | ECT3896 (Port Aransas Jetty, Texas) | KC309482, KC309554, KC309624 |
| *Navicula cari* Ehrenberg |  |  | AM501991, AM710457, N/A |
| *Navicula cryptocephala* Kützing | HK090 | FD109 (UTEX) | HQ912603, HQ912467, HQ912296 |
| *Navicula hippodontofallax* Witkowski & Ch. Li | SZCZCH703 |  | KT943636, KT943661, KT943695 |
| *Navicula perminuta* Østrup | mbccc3 |  | JQ045340, JQ432375, N/A |
| *Navicula sp.* Bory | HK486 | Coz4 (Cozumel, Mexico) | MH040322, MH040271, MH040244 |
| *Navicula sp.* Bory | HK487 | Coz4 (Cozumel, Mexico) | N/A, MH064103, MH064010 |
| *Navicula sp.* Bory | HK488 | 24IV14-2A (Conch Reef, Florida) | MH063475, MH064104, MH064011 |
| *Navicula sp.* Bory | HK489 | 24IV14-3A (Pickles Reef, Florida) | MH063476, MH064105, MH064012 |
| *Navicula sp.* Bory | HK490 | GU7Y-4 (University of Guam Marine Laboratories, Guam) | N/A, MH040272, MH040245 |
| *Navicula sp.* Bory | KSA0112 | SA23 (Al-Wajh, Saudi Arabia) | N/A, MH064106, MH064013 |
| *Navicula sp.* Bory | UTKSA0131 | KSA2015-19 (Al-Nawras, Jeddah, Saudi Arabia) | MH063477, MH064107, MH064014 |
| *Navicula sp.* Bory | UTKSA0162 | KSA2015-14 (Bhadur Resort, Saudi Arabia) | MH063478, MH064108, MH064015 |
| *Navicula sp.* Bory | UTKSA0239 | KSA2015-41 (Rabigh, Saudi Arabia) | MH063479, MH064109, MH064016 |
| *Navicula reinhardtii* Grunow in Cleve & Möller |  |  | AM501976, AM710442, N/A |
| *Navicula tripunctata* (O.F. Müller) Bory |  |  | AM502028, AM710495, N/A |
| *Navicula zhengii* Witkowski & Li | SZCZCH96 |  | KT943632, KT943681, KT943691 |
| *Neidium affine* (Ehrenberg) Pfitzer | HK064 | FD127 (UTEX) | HQ912583, HQ912447, HQ912276 |
| *Neidium bisulcatum* (Lagerstedt) Cleve | HK076 | FD417 (UTEX) | HQ912591, HQ912455, HQ912284 |
| *Neidium productum* (W. Smith) Cleve | HK063 | FD116 (UTEX) | HQ912582, HQ912446, HQ912275 |
| *Nitzschia acidoclinata* Lange-Bertalot |  |  | KC736632, KC736602, N/A |
| *Nitzschia aurariae* Cholnoky | SZCZCH966 |  | KT943639, KT943663, KT943698 |
| *Nitzschia cf dissipata* (Kützing) Rabenhorst | KSA0035 | SA4 (Durrah, Saudi Arabia) | KU179128, KU179116, KU179143 |
| *Nitzschia draveillensis* Coste & Ricard |  |  | KC736635, KC736605, N/A |
| *Nitzschia dubiformis* Hustedt |  |  | AB430616, AB430696, N/A |
| *Nitzschia inconspicua* Grunow |  |  | KC736636, KC736607, N/A |
| *Nitzschia filiformis* (W. Smith) Van Heurck | HK073 | FD267 (UTEX) | HQ912589, HQ912453, HQ912282 |
| *Nitzschia cf frigida* Grunow | HK468 | AKIce (Barrow, Alaska) | N/A, MH064110, MH064017 |
| *Nitzschia frustulum* (Kützing) Grunow | TCC545 |  | KT072974, KT072922, N/A |
| *Nitzschia cf longissima* (Brébisson in Kützing) Grunow | HK176 | ECT3689 (Sala Glula, Guam) | KX981850, KX981829, KX981804 |
| *Nitzschia longissima* (Brébisson in Kützing) Grunow | GenBank |  | AY881968, AY881967, N/A |
| *Nitzschia longissima* (Brébisson in Kützing) Grunow | UTKSA0021 | SA29 (Jeddah, Saudi Arabia) | MH063480, MH064111, MH064018 |
| *Nitzschia longissima* (Brébisson in Kützing) Grunow | UTKSA0124 | KSA2015-9 (Bhadur Resort, Saudi Arabia) | MH063481, MH064112, MH064019 |
| *Nitzschia lorenziana* Grunow |  |  | KC736637, KC736608, N/A |
| *Nitzschia martiana* (C. Agardh) Van Heurck | HK405 | 3VIII07 (Talofofo Bay, Guam) | N/A, KJ577899, KJ577933 |
| *Nitzschia sp.* Hassall | KSA0120 | SA27 (Jeddah, Saudi Arabia) | KX981849, KX981828, KX981803 |
| *Nitzschia sp.* Hassall | HK469 | Rincon Mangrove (Costa Rica) | MH040323, MH040273, MH040246 |
| *Nitzschia sp.* Hassall | HK470 | Nate Site 1 (Kona, Hawaii) | MH040324, MH040274, MH040247 |
| *Nitzschia sp.* Hassall | HK472 | Coz4 (Cozumel, Mexico) | MH040325, N/A, MH040248 |
| *Nitzschia sp.* Hassall | HK473 | GU52X-4 (Outhouse Beach, Guam) | MH040326, MH040275, MH040249 |
| *Nitzschia sp.* Hassall | HK474 | CCMP1698 (NCMA) | MH040327, MH040276, MH040250 |
| *Nitzschia sp.* Hassall | UTKSA0053 | SA19 (Al-Wajh, Saudi Arabia) | N/A, MH064113, MH064020 |
| *Nitzschia sp.* Hassall | UTKSA0102 | KSA2015-14 (Bhadur Resort, Saudi Arabia) | MH063482, MH064114, MH064021 |
| *Nitzschia sp.* Hassall | UTKSA0106 | KSA2015-49 (Duba, Saudi Arabia) | MH063483, MH064115, MH064022 |
| *Nitzschia sp.* Hassall | UTKSA0107 | KSA2015-49 (Duba, Saudi Arabia) | MH063484, MH064116, MH064023 |
| *Nitzschia sp.* Hassall | UTKSA0109 | KSA2015-16 (Al-Nawras, Jeddah, Saudi Arabia) | MH063485, MH064117, N/A |
| *Nitzschia sp.* Hassall | UTKSA0111 | KSA2015-23 (Markaz Al Shoaibah, Saudi Arabia) | MH063486, MH064118, MH064024 |
| *Nitzschia sp.* Hassall | UTKSA0171 | KSA2015-11 (Bhadur Resort, Saudi Arabia) | MH063487, MH064119, MH064025 |
| *Nitzschia sp.* Hassall | UTKSA0173 | KSA2015-37 (Rabigh, Saudi Arabia) | MH063488, MH064120, MH064026 |
| *Nitzschia sp.* Hassall | UTKSA0182 | KSA2015-38 (Rabigh, Saudi Arabia) | MH063489, MH064121, MH064027 |
| *Nitzschia sp.* Hassall | UTKSA0260 | KSA2015-11 (Bhadur Resort, Saudi Arabia) | MH063490, MH064122, MH064028 |
| *Nitzschia cf spathulata* Brébisson ex W.Smith | UTKSA0332 | KSA2016-1 (Bhadur Resort, Saudi Arabia) | N/A, MH064123, MH064029 |
| *Nitzschia cf spathulata* Brébisson ex W.Smith | UTKSA0387 | KSA2016-35 (Duba, Saudi Arabia) | MH063491, MH064124, MH064030 |
| *Nitzschia traheaformis* Ch. Li, Witkowski & Yu Sh. | SZCZCH970 |  | KT943642, KT943666, KT943701 |
| *Nitzschia traheaformis* Ch. Li, Witkowski & Yu Sh. | SZCZCH971 |  | KT943643, KT943667, KT943702 |
| *Nitzschia volvendirostrata* Ashworth, Dabek & Witkowski | KSA0039 | SA12 (Markaz Al Shoaibah, Saudi Arabia) | N/A, KU179112, KU179139 |
| *Parlibellus hamulifer* (Grunow) Cox | HK409 | GU44AK-4 (Gab Gab Beach, Guam) | KJ577866, KJ577903, KJ577937 |
| *Parlibellus* cf *hamulifer* (Grunow) Cox | HK428 | SantaRosaCor.green (Costa Rica) | KU179137, KU179122, KU179149 |
| *Parlibellus harffianus* Witkowski, Ch. Li & S.-X.Yu | SZCZCH75 |  | KT943652, KT943686, KT943715 |
| *Phaeodactylum tricornutum* Bohlin | HK011 | CCMP2561 (NCMA) | HQ912556, HQ912420, HQ912250 |
| *Phaeodactylum tricornutum* Bohlin | HK538 | UTEX640 (UTEX) | MH063492, MH064125, MH064031 |
| *Phaeodactylum tricornutum* Bohlin | HK539 | UTEX646 (UTEX) | MH063493, MH064126, MH064032 |
| *Phaeodactylum tricornutum* Bohlin | HK540 | UTEX2089 (UTEX) | MH063494, MH064127, MH064033 |
| *Pinnularia brebissonii* (Kützing) Rabenhorst | HK092 | FD274 (UTEX) | HQ912604, HQ912468, HQ912297 |
| *Pinnularia termitina* (Ehrenberg) Patrick | HK088 | FD484 (UTEX) | HQ912601, HQ912465, HQ912294 |
| *Placoneis elginensis* (Gregory) Cox | HK096 | FD416 (UTEX) | HQ912607, HQ912471, HQ912300 |
| *Plagiotropis sp.* Pfitzer | HK508 | PR5 (Condado Lagoon, Puerto Rico) | MH063495, MH064128, MH064034 |
| *Planothidium frequentissimum* (Lange-Bertalot) Lange-Bertalot | PF1 |  | KJ658409, KJ658392, N/A |
| *Planothidium lanceolatum* (Brébisson ex Kützing) Lange-Bertalot | PL2 |  | KJ658410, KJ658393, N/A |
| *Planothidium sp.* Round & Bukhtiyarova | SZCZCH26 |  | KT943653, KT943678, KT943716 |
| *Pleurosigma sp.* W. Smith | HK495 | GU52X-1 (Outhouse Beach, Guam) | MH040327, MH040276, MH040250 |
| *Pleurosigma sp.* W. Smith | UTKSA0019 | SA18 (Duba, Saudi Arabia) | KX981840, KX981822, KX981798 |
| *Pleurosigma sp.* W. Smith | UTKSA0167 | KSA2015-49 (Duba, Saudi Arabia) | MH063496, MH064129, MH064035 |
| *Pleurosigma sp.* W. Smith | UTKSA0264 | KSA2015-16 (Al-Nawras, Jeddah, Saudi Arabia) | MH063497, N/A, MH064036 |
| *Pleurosigma sp.* W. Smith | UTKSA0273 | KSA2015-16 (Al-Nawras, Jeddah, Saudi Arabia) | MH063498, MH064130, MH064037 |
| *Pleurosigma stuxbergii* Cleve & Grunow | SZCZCH973 |  | N/A, KT943674, KT943711 |
| *Psammodictyon constrictum* (Gregory) Mann in Round, Crawford & Mann | HK440 | GU7X-7 (University of Guam Marine Lab, Guam) | KX981851, KX981830, KX981805 |
| *Psammodictyon constrictum* (Gregory) Mann in Round, Crawford & Mann | HK471 | Nate Site 1 (Kona, Hawaii) | MH040329, MH040278, MH040252 |
| *Psammodictyon sp.* D.G. Mann | UTKSA0117 | KSA2015-30 (Markaz Al Shoaibah, Saudi Arabia) | MH063499, MH064131, MH064038 |
| *Psammodictyon sp.* D.G. Mann | UTKSA0151 | KSA2015-37 (Rabigh, Saudi Arabia) | MH063500, MH064132, MH064039 |
| *Psammodictyon sp.* D.G. Mann | UTKSA0280 | KSA2015-2 (Bhadur Resort, Saudi Arabia) | MH063501, MH064133, MH064040 |
| *Psammodictyon pustulatum* (Voigt ex Meister) Lobban | UTKSA0298 | KSA2015-38 (Rabigh, Saudi Arabia) | MH063502, MH064134, MH064041 |
| *Rhoiconeis pagoensis* C.S. Lobban | HK419 | GU7X-7 (University of Guam Marine Lab, Guam) | KX981846, KX981825, KX981800 |
| *Rhoiconeis sp.* Grunow | UTKSA0128 | KSA2015-16 (Al-Nawras, Jeddah, Saudi Arabia) | MH063503, MH064135, N/A |
| *Rhoicosigma sp.* Grunow | UTKSA0194 | KSA2015-22 (Markaz Al Shoaibah, Saudi Arabia) | MH063504, MH064136, MH064042 |
| *Rhoicosphenia abbreviata* (C.Agardh) Lange-Bertalot | CH030 |  | KJ011672, KJ011854, N/A |
| *Rhoicosphenia cf abbreviata* (C.Agardh) Lange-Bertalot | EWT2016.80 |  | KU965569, KU965580, N/A |
| *Rhopalodia contorta* Hustedt |  | L1299 (UTEX) | HQ912406, HQ912392, HQ912378 |
| *Rhopalodia gibba* (Ehrenberg) O. Müller |  |  | HQ912407, HQ912393, HQ912379 |
| *Rhopalodia sp.* O. Müller | HK433 | 21IV14-4D (Rabbit Key Basin, Florida) | KX981843, KX981823, KX981799 |
| *Rhopalodia sp.* O. Müller |  | ECT3678 (Tinago River, Guam) | HQ912405, HQ912391, HQ912377 |
| *Rossia sp.* Voigt |  |  | EF151968, EF143281, N/A |
| *Schizostauron sp.* Grunow | UTKSA0141 | KSA2015-11 (Bhadur Resort, Saudi Arabia) | MH063505, MH064137, MH064043 |
| *Schizostauron sp.* Grunow | SZCZP32 |  | KT943595, KT943606, KT943619 |
| *Schizostauron sp.* Grunow | SZCZP40 |  | KT943596, KT943607, KT943620 |
| *Scoliopleura peisonis* Grunow | HK103 | FD13 (UTEX) | HQ912609, HQ912473, HQ912302 |
| *Sellaphora laevissima* (Kützing) D.G.Mann | THR4 |  | EF151981, EF143309, N/A |
| *Sellaphora minima* Grunow | TCC524 |  | KF959656, KF959642, N/A |
| *Sellaphora seminulum* (Grunow) D.G. Mann | TCC461 |  | KF959642, KC736613, N/A |
| *Seminavis robusta* D.B.Danielidis & D.G.Mann | HK492 | GU7X-7 (University of Guam Marine Laboratories, Guam) | MH040330, MH040279, MH040253 |
| *Stauroneis acuta* W. Smith | HK059 | FD51 (UTEX) | HQ912579, HQ912443, HQ912272 |
| *Stauroneis anceps* Ehrenberg |  |  | AM502008, AM710475, N/A |
| *Stauroneis gracilior* Reichardt |  |  | AM501988, AM710454, N/A |
| *Stauroneis kriegeri* Patrick |  |  | AM501990, AM710456, N/A |
| *Stauroneis phoenicentron* (Nitzsch) Ehrenberg |  |  | AM502031, AM710498, N/A |
| *Stauroneis sp.* Ehrenberg | UTKSA0410 | KSA2016-9 (Bhadur Resort, Saudi Arabia) | MH063506, MH064138, MH064044 |
| *Sternimirus shandongensis* Witkowski & Li | SZCZCH968 |  | KT943637, KT943662, KT943696 |
| *Staurotropis americana* Ashworth | HK442 | FishPassMangrove (Mustang Island, Texas) | KX981855, KX981834, KX981808 |
| *Staurotropis americana* Ashworth | HK443 | Coz4 (Cozumel, Mexico) | KX981854, KX981833, KX981807 |
| *Staurotropis khiyamii* J. Sabir & Ashworth | UTKSA0047 | SA18 (Duba, Saudi Arabia) | KX981853, KX981832, KX981806 |
| *Staurotropis seychellensis* (Giffen) Paddock | HK172 | ECT3721 (University of Guam Marine Lab, Guam) | KX981856, N/A, KX981809 |
| *Stenopterobia curvula* (W. Smith) Krammer |  | L541 (UTEX) | HQ912416, HQ912402, HQ912388 |
| *Surirella cf fastuosa* (Ehrenberg) Ehrenberg |  | SZCZCH189 | KT943629, KT943655, KT943688 |
| *Surirella minuta* Van Heurck |  | FD320 (UTEX) | HQ912658, HQ912522, HQ912351 |
| *Surirella ovata* Kützing | HK214 | L1241 (UTEX) | HQ912658, HQ912522, HQ912351 |
| *Surirella splendida* (Ehrenberg) Kützing |  |  | HQ912415, HQ912401, HQ912387 |
| *Surirella sp.* Turpin | UTKSA0299 | KSA2015-2 (Bhadur Resort, Saudi Arabia) | MH063507, MH064139, MH064045 |
| *Tetramphora chilensis* (Hustedt) Stepanek & Kociolek |  | AMPH132 | KU665638, KU665639, KU665640 |
| *Trachyneis sp.* P.T. Cleve | HK439 | SantaRosaCor.green (Costa Rica) | KX981845, KX981824, N/A |
| *Tryblionella apiculata* Gregory | HK087 | FD465 (UTEX) | HQ912600, HQ912464, HQ912293 |
| *Tryblionella gaoana* Witkowski & Ch. Li | SZCZCH97 |  | KT943638, KT943683, KT943697 |
| unidentified diploneid | UTKSA0368 | KSA0216-36 (Duba, Saudi Arabia) | MH063508, MH064140, MH064046 |
| unidentified monoraphid | HK380 | ECT3899 (Pacific Grove, California) | KJ577839, KJ577874, KJ577911 |
| unidentified monoraphid | HK427 | BallenaEstRock (Costa Rica) | KU179136, KU179121, KU179148 |
| unidentified monoraphid | UTKSA0152 | KSA2015-37 (Rabigh, Saudi Arabia) | MH063509, MH064141, MH064047 |
| unidentified monoraphid | UTKSA0158 | KSA2015-37 (Rabigh, Saudi Arabia) | MH063510, MH064142, N/A |
| unidentified naviculoid | HK497 | 23X15-5B (Harbor Branch Oceanographic Institute boat launch) | MH063511, MH064143, MH064048 |
| unidentified naviculoid | UTKSA0247 | KSA2015-5 (Bhadur Resort, Saudi Arabia) | MH063512, MH064144, MH064049 |
| unidentified stauroneid | UTKSA0220 | KSA2015-7 (Bhadur Resort, Saudi Arabia) | MH063513, MH064145, MH064050 |
| Araphid Outgroups |  |  |  |
| *Asterionella formosa* Hassall | HK144 | UTCC605 | HQ912633, HQ912497, HQ912326 |
| *Asterionellopsis glacialis* (Castracane) Round | HK107 | CCMP134 (NCMA) | HQ912613, HQ912477, HQ912306 |
| *Asterionellopsis socialis* (Lewin & Norris) Crawford & Gardner | HK181 | CCMP1717 (NCMA) | HQ912646, HQ912510, HQ912339 |
| *Asterionellopsis socialis* (Lewin & Norris) Crawford & Gardner | HK319 | ECT3920 (Ft. Stevens State Park, Oregon) | JX413545, JX413562, JX413579 |
| *Astrosyne radiata* Ashworth & Lobban | HK169 | ECT3697 (Gab Gab Beach, Guam) | JN975238, JN975252, JN975267 |
| *Bleakeleya notata* (Grunow in Van Heurck) F.E. Round | HK247 | ECT3733 (Pago Bay, Guam) | HM627330, HM627327, HM627324 |
| *Castoridens hyalina* Ashworth, Witkowski & Li | HK444 | C1 12-7-13 (Destin-Choctawhatchee Bay, Florida) | N/A, KU851892, KU851907 |
| *Castoridens striata* Ashworth, Li & Witkowski | HK385 | 15VI11-2A (Baffin Bay, Texas) | KJ577844, KJ577879, KJ577915 |
| *Catacombas gaillonii* (Bory de Saint-Vincent) Williams & Round | s0045 |  | KR048195, KR048217, KR048229 |
| *Centronella reicheltii* Voigt | HK150 | CCAP1011/1 | HQ912635, HQ912499, HQ912328 |
| *Ctenophora pulchella* (Ralfs ex Kützing) Williams & Round | HK105 | FD150 (UTEX) | HQ912611, HQ912475, HQ912304 |
| *Cyclophora castracanei* Ashworth & Lobban | HK243 | GU44AB-6 (Gab Gab Beach, Guam) | JN975242, JN975256, JN975271 |
| *Cyclophora castracanei* Ashworth & Lobban | HK395 | GU44AN-7 (Gab Gab Beach, Guam) | KJ577854, KJ577889, N/A |
| *Cyclophora cf minor* Ashworth & Lobban | HK461 | 24IV14-3A (Pickles Reef, Florida) | MH040308, MH040254, MH040230 |
| *Cyclophora tabellariformis* Ashworth & Lobban | HK306 | ECT3892 (Carrabelle, Florida) | JN975243, JN975257, JN975272 |
| *Cyclophora tabellariformis* Ashworth & Lobban | HK460 | GU44AY-6 (Gab Gab Beach, Guam) | MH040309, MH040255, N/A |
| *Cyclophora tenuis* Castracane | HK216 | ECT3723 (Umatac Bay, Guam) | HQ912660, HQ912524, HQ912353 |
| *Cyclophora tenuis* Castracane | HK307 | ECT3854 (Kahana Beach Park, Oahu, Hawaii) | JN975240, JN975254, JN975269 |
| *Cyclophora tenuis* Castracane | HK308 | ECT3838 (Long Beach, California) | JN975241, JN975255, JN975270 |
| *Delphineis surirella* (Ehrenberg) G.W. Andrews | HK133 | CCMP1095 | HQ912629, HQ912493, HQ912322 |
| *Delphineis surirella* (Ehrenberg) G.W. Andrews | HK295 | ECT3886 (Bald Head Island, North Carolina) | JX413544, JX413561, JX413578 |
| *Diatoma elongata* (Lyngbye) C.Agardh | HK119 | UTCC62 | HQ912622, HQ912486, HQ912315 |
| *Diatoma tenue* Agardh | HK078 | FD106 (UTEX) | HQ912593, HQ912457, HQ912286 |
| *Dimeregramma sp.* J. Ralfs in A. Pritchard | HK288 | ECT3864 (MSI, Port Aransas, Texas) | JN975244, JN975258, JN975273 |
| *Dimeregramma sp.* J. Ralfs in A. Pritchard | HK358 | 15VI11-2A (Baffin Bay, Texas) | JX401231, JX401249, JX401267 |
| *Dimeregramma sp.* J. Ralfs in A. Pritchard | HK359 | ECT3891 (St. George Island, Florida) | JX401232, JX401250, JX401268 |
| *Dimeregramma sp.* J. Ralfs in A. Pritchard | HK376 | 25VI12-1C (Hunting Island, South Carolina) | KF701596, KF701605, KF701614 |
| *Dimeregramma sp.* J. Ralfs in A. Pritchard | HK377 | AtlanticPlankton#8 (Florida) | KF701597, KF701606, KF701615 |
| *Florella pascuensis* Navarro | HK175 | ECT3756 (Guam) | JN975246, JN975260, JN975275 |
| *Fragilariforma virescens* (Ralfs) Williams & Round | HK132 | FD291 (UTEX) | HQ912628, HQ912492, HQ912321 |
| *Glyphodesmis sp.* Greville | HK357 | ECT3891 (St. George Island, Florida) | N/A, JX401248, JX401266 |
| *Grammatophora macilenta* W. Smith | HK368 | GU44AK-4 (Gab Gab Beach, Guam) | JX401241, JX401259, JX401276 |
| *Grammatophora oceanica* Ehrenberg | HK147 | CCMP410 | HQ912634, HQ912498, HQ912327 |
| *Grammatophora sp.* Ehrenberg | HK459 | Nate Site 1 (Hawaii) | MG684352, MG684323, MG684295 |
| *Grammatophora sp.* Ehrenberg | UTKSA0132 | KSA2015-16 (Al-Nawras, Jeddah, Saudi Arabia) | MH063514, MH064146, MH064051 |
| *Grammatophora undulata* Ehrenberg | HK367 | Coz-3 (Cozumel, Mexico) | JX401240, JX401258, JX401275 |
| *Grammonema striatula* (Lyngbye) Agardh | HK371 | ECT3897 (Pebble Beach, California) | KF701591, KF701600, KF701609 |
| *Hanicella moenia* Lobban & Ashworth | HK379 | GU44AK-6 (Gab Gab Beach, Guam) | KF701599, KF701608, KF701617 |
| *Hendeyella dimeregrammopsis* Ashworth | HK391 | Coz-1 (Cozumel, Mexico) | KJ577850, KJ577885, KJ577920 |
| *Hendeyella lineata* Ashworth & Lobban | HK325 | GU44AI-3 (Gab Gab Beach, Guam) | JX413547, JX413564, JX413581 |
| *Koernerella recticostata* (Körner) Ashworth, Lobban & Theriot | HK242 | GU44AB-8 (Gab Gab Beach, Guam) | HM627331, HM627328, HM627325 |
| *Licmophora abbreviata* Agardh | UTKSA0049 | SA29 (Jeddah, Saudi Arabia) | KP125882, KP125883, KP125884 |
| *Licmophora colosalis* Belando, Aboal & Jiménez | HK366 | ECT3907 (Rabbit Key Basin, Florida) | JX401239, JX401257, JX401274 |
| *Licmophora colosalis* Belando, Aboal & Jiménez | UTKSA0066 | SA29 (Jeddah, Saudi Arabia) | MG684358, MG684329, MG684299 |
| *Licmophora aff ehrenbergii* (Kützing) Grunow | HK420 | GU7X-6 (University of Guam Marine Lab, Guam) | KP125876, KP125879, KP125881 |
| *Licmophora flucticulata* Lobban, Schefter & Ruck |  | GU56-A (Cocos Wall, Guam) | HQ997923, JN975262, JN975277 |
| *Licmophora normaniana* (Greville) Wahrer in Wahrer, Fryxell & Cox | HK403 | 26II12-1 (Mustang Island, Texas) | KJ577860, KJ577897. KJ577931 |
| *Licmophora paradoxa* (Lyngbye) Agardh | HK106 | CCMP2313 | HQ912612, HQ912476, HQ912305 |
| *Licmophora peragallioides* (Lobban) Lobban & Ashworth | HK364 | GU44AL-3 (Gab Gab Beach, Guam) | JX401237, JX401255, JX401273 |
| *Licmophora cf remulus* Grunow | HK302 | GU52-O (Outhouse Beach, Guam) | JN975248, JN975263, N/A |
| *Licmophora sp.* Agardh | HK365 | Coz-2 (Cozumel, Mexico) | JX401238, JX401256, N/A |
| *Licmophora sp.* Agardh | KSA0085 | SA4 (Durrah, Saudi Arabia) | MG684353, MG684324, N/A |
| *Licmophora sp*. Agardh | KSA0151 | SA1 (Durrah, Saudi Arabia) | MG684354, MG684325, N/A |
| *Licmophora sp*. Agardh | UTKSA0010 | SA29 (Jeddah, Saudi Arabia) | MG684355, MG684326, MG684296 |
| *Licmophora sp*. Agardh | UTKSA0029 | SA18 (Duba, Saudi Arabia) | MG684356, MG684327, MG684297 |
| *Licmophora sp*. Agardh | UTKSA0050 | SA18 (Duba, Saudi Arabia) | MG684357, MG684328, MG684298 |
| *Licmophora sp*. Agardh | UTKSA0084 | SA18 (Duba, Saudi Arabia) | MG684359, MG684330, MG684300 |
| *Licmophora sp*. Agardh | UTKSA0191 | KSA2015-14 (Bhadur Resort, Saudi Arabia) | MH063515, MH064147, MH064052 |
| *Lucanicum concatenatum* Lobban & Ashworth | HK378 | GU44AI-3 (Gab Gab Beach, Guam) | KF701598, KF701607, KF701616 |
| *Microtabella interrupta* (Ehrenberg) Round | HK248 | ECT3700 (Gab Gab Beach, Guam) | JN975247, JN975261, JN975276 |
| *Microtabella interrupta* (Ehrenberg) Round | HK458 | 20X15-1 (Boca Chica Channel, Florida) | MH040319, MH040265, MH040238 |
| *Nanofrustulum cf shiloi* (J.J. Lee, C.W. Reimer, & M.E. McEnery) F.E. Round, H. Hallsteinsen, & E. Paasche | HK056 | CCMP2649 | HQ912578, HQ912442, HQ912271 |
| *Neodelphineis sp.* Takano | HK421 | FijiBottleNY (New York) | KP125875, KP125878, N/A |
| *Neofragilaria nicobarica* Desikachary, Prasad & Prema | s0371 |  | AB433340, KR048216 KR048228 |
| *Neofragilaria cf nicobarica* Desikachary, Prasad & Prema | HK375 | Coz-1 (Cozumel, Mexico) | KF701595, KF701604, KF701613 |
| *Neosynedra provincialis* (Grunow) Williams & Round | HK457 | 24IV14-3A (Pickles Reef, Florida) | N/A, MH040266, MH040239 |
| *Opephora guenter-grassi* (Witkowski & Lange-Bertalot) Sabbe & Vyverman |  | s0263 | AB436781, KR048218, N/A |
| *Opephora pacifica* (Grunow) Petit | HK296 | ECT3831 (Ward Island, Texas) | JN975249, JN975264, JN975278 |
| *Perideraion elongatum* Jordan, Arai & Lobban | HK411 | GU44AK-6 (Gab Gab Beach, Guam) | KJ577868, KJ577905, KJ577939 |
| *Perideraion cf elongatum* Jordan, Arai & Lobban | UTKSA0259 | KSA2015-49 (Duba, Saudi Arabia) | MH063516, MH064148, MH064053 |
| *Perideraion montgomeryii* Lobban, Jordan & Ashworth | HK246 | GU7 (University of Guam Marine Lab, Guam) | HM627332, HM627329, HM627326 |
| *Plagiogramma sp.* Greville | HK212 | ECT3776 (Taeleyag Beach, Guam) | HQ912656, HQ912520, HQ912349 |
| *Plagiogramma sp.* Greville | HK324 | ECT3924 (Potlatch State Park, Washington) | JX413546, JX413563, JX413580 |
| *Plagiogramma sp.* Greville | HK374 | 25VI12-1C (Hunting Island, South Carolina) | KF701594, KF701603, KF701612 |
| *Plagiostriata goreensis* Sato & Medlin | s0388 |  | KR048198, KR048220, KR048232 |
| *Psammogramma vigoensis* Sato & Medlin | s0391 |  | KR048194, KR048215, KR048227 |
| *Psammoneis japonica* Sato, Kooistra & Medlin | HK299 | GU52-O (Outhouse Beach, Guam) | JN975250, JN975265, JN975279 |
| *Psammoneis obaidii* Ashworth & Sabir | UTKSA0057 | SA12 (Markaz Al Shoaibah, Saudi Arabia) | KR059023, KR059022, KR059024 |
| *Psammoneis sp.* Sato, Kooistra & Medlin | UTKSA0250 | KSA2015-42 (Rabigh, Saudi Arabia) | MH063517, MH064149, N/A |
| *Psammotaenia lanceolata* Ashworth, Li & Witkowski | HK316 | 10X10-2 (St. George Island, Florida) | JX413543, JX413560, JX413577 |
| *Pseudostriatella oceanica* Sato, Mann & Medlin | s0384 |  | KR048197, KR048219, KR048231 |
| *Pteroncola sp.*R.W. Holmes & D.A. Croll | UTKSA0078 | SA29 (Jeddah, Saudi Arabia) | MG684376, N/A, MG684316 |
| *Podocystis cf americana* Bailey | HK453 | 19X15-1A (Channel #5, Florida) | MH040320, MH040267, MH040240 |
| *Podocystis cf americana* Bailey | HK454 | 19X15-1B (Channel #5, Florida) | MG684360, MG684331, MG684301 |
| *Podocystis spathulata* (Shadbolt) Van Heurck | HK217 | ECT3733 (Pago Bay, Guam) | HQ912661, HQ912525, HQ912354 |
| *Rhabdonema adriaticum* Kützing | HK370 | Coz-3 (Cozumel, Mexico) | JX401243, JX401261, JX401278 |
| *Rhabdonema arcuatum* (Lyngbye) Kützing | HK304 | ECT3898 (Pebble Beach, California) | JN975251, JN975266, JN975280 |
| *Rhabdonema sp.* Kützing | HK369 | GU44AI-1 (Gab Gab Beach, Guam) | JX401242, JX401260, JX401277 |
| *Rhaphoneis amphiceros* (Ehrenberg) Ehrenberg | HK237 | ECT3828 (Redfish Bay, Texas) | HQ912673, HQ912537, KC309625 |
| *Rhaphoneis amphiceros* (Ehrenberg) Ehrenberg | HK373 | 25VI12-1A (Hunting Island, South Carolina) | KF701593, KF701602, KF701611 |
| *Serratifera varisterna* Li, Ashworth & Witkowski | HK315 | 9X10-2 (Florida State University Marine Lab, Florida) | JX413542, JX413559, JX413576 |
| *Serratifera varisterna* Li, Ashworth & Witkowski | HK424 | PackaryChannelPlankton (Mustang Island, Texas) | KU851868, KU851879, KU851894 |
| *Staurosira construens* Ehrenberg | HK071 | FD232 (UTEX) | HQ912587, HQ912451, HQ912280 |
| *Staurosirella pinnata* (Ehrenberg) Williams & Round | HK116 | CCMP330 (NCMA) | HQ912620, HQ912484, HQ912313 |
| *Striatella unipunctata* (Lyngbye) Agardh | HK177 | ECT3648 (Asan Beach, Guam) | HQ912643, HQ912507, HQ912336 |
| *Striatella unipunctata* (Lyngbye) Agardh | HK318 | ECT3874 (Channel #5, Florida) | JX419383, JX419384, JX419385 |
| *Stricosus blumbergii* Theriot & Ashworth | HK362 | 15VI11-2A (Baffin Bay, Texas) | JX401235, JX401253, JX401271 |
| *Stricosus harrisonii* Lobban & Theriot | HK363 | GU44AI (Gab Gab Beach, Guam) | JX401236, JX401254, JX401272 |
| *Synedra famelica* Kützing | HK072 | FD255 (UTEX) | HQ912588, HQ912452, HQ912281 |
| *Synedra ulna* (Nitzsch) Ehrenberg | HK075 | FD404 (UTEX) | HQ912590, HQ912454, HQ912283 |
| *Synedropsis hyperborea* (Grunow) Hasle, Medlin & Syvertsen | HK117 | CCMP1423 (NCMA) | HQ912621, HQ912485,  HQ912314 |
| *Synedropsis cf recta* Hasle, Medlin & Syvertsen | HK110 | CCMP1620 (NCMA) | HQ912616, HQ912480, HQ912309 |
| *Tabellaria flocculosa* (Roth) Kützing | HK065 | FD133 (UTEX) | HQ912584, HQ912448, HQ912277 |
| *Tabularia cf tabulata* (Agardh) Snoeijs | HK109 | CCMP846 (NCMA) | HQ912615, HQ912479, HQ912308 |
| *Talaroneis posidoniae* Kooistra & De Stefano | WK59 |  | AY216905, KR048214, KR048226 |
| *Tetracyclus sp*. Ralfs | HK416 | B12 (Lake Baikal, Russia) | KJ577873, KJ577910, KJ577944 |
| *Thalassionema cf bacillare* (Heiden) Kolbe | HK361 | ECT3929 (Gulf of Mexico, Texas) | JX401234, JX401252, JX401270 |
| *Thalassionema frauenfeldii* (Grunow) Tempère & Peragallo | HK372 | 25VI12-1A (Hunting Island, South Carolina) | KF701592, KF701601, KF701610 |
| *Thalassionema cf nitzschioides* (Grunow) Mereschkowsky | HK360 | ECT3929 (Gulf of Mexico, Texas) | JX401233, JX401251, JX401269 |
